# Entering Kindergarten After Years of Play: A Cross-Case Analysis of School Readiness Following Play-Based Education

**DOI:** 10.1007/s10643-022-01428-w

**Published:** 2022-11-15

**Authors:** Lisa Fyffe, Pat L. Sample, Angela Lewis, Karen Rattenborg, Anita C. Bundy

**Affiliations:** 1 Department of Occupational Therapy, Colorado State University, Fort Collins, CO 80523 USA; 2 School of Education, Colorado State University, Fort Collins, CO 80523 USA; 3 Department of Human Development and Family Studies, Colorado State University, Fort Collins, CO USA

**Keywords:** School readiness, Play-based education, Reggio Emilia educational philosophy, Kindergarten transition, Early childhood development

## Abstract

Cross-case study research was used to explore the school readiness of four 5-year-old children entering kindergarten during the 2020–2021 school year after three or more years of play-based early childhood education at a Reggio Emilia-inspired early childhood education center. Data included a series of three 1-h individual interviews with four mothers and three kindergarten teachers, field visits during remote learning, and artifact collection over the course of the school year. Themes describing the children’s school readiness were developed through cross-case analysis. Participants described the children as learners, explorers, communicators, and empathizers. The learner theme centers on the children’s responsiveness to instruction; the explorer theme describes how the children approached learning; the communicator theme illustrates the children’s prowess with social connection and self-advocacy, and the empathizer theme shows the thoughtfulness and emotional sensitivity these children displayed. Findings suggest that play-based learning prepared these children for successful kindergarten experiences and was a viable early childhood education pedagogy fostering school readiness.

## Introduction

School readiness is a culmination of a lifetime of experiences that prepare children to enter a group learning context where they must modify their actions in response to feedback, establish relationships with peers and adults, and apply new knowledge within a variety of learning contexts. Thus, children benefit from formal education when they have developed processes that support learning, such as establishing social relationships, self-management, and positive approaches to learning (Eggum-Wilkins et al., 2014; Ginsburg, 2007; Pistorova & Slutsky, 2018).

The National Association for the Education of Young Children (NAEYC, 2017) promotes the use of play as an educational pedagogy during early childhood to facilitate the social adaptation, inquisitiveness, and self-regulation necessary for comprehending academic content and general knowledge. NAEYC (2017) described play in early childhood education as “a valuable pedagogical tool in that it features the precise contexts that facilitate learning… mental activation, engagement, social interaction, and meaningful connections” (p. 3). Play has been considered the foundation of children’s learning dating back to the days of Plato (427–347 BC) and Aristotle (387–322 BC), both of whom wrote about the virtues of play as necessary to develop children into competent adults. Vygotsky (1978) described that exploring culturally instilled roles through play developed attention, memory, abstract thought, and self-monitoring. Elkonin (1978) extended this discussion by saying that play fostered children’s mental representation, motivation and intentionality, awareness of multiple perspectives, behavior modification in accordance with social norms, and promotes internal morality as children create, follow, adapt, and enforce rules.

More recently, the American Academy of Pediatrics (Ginsburg, 2007) described play as “essential to the cognitive, physical, social and emotional well-being of children and youth” and called for “the inclusion of play as we... prepare our children to be academically, socially, and emotionally equipped to lead us into the future” (p. 183). Zosh et al. (2017) concurred, stating that “content only serves children as far as they can apply and build on it… children also need a deep conceptual understanding to connect concepts and skills, apply knowledge and spark new ideas” (p. 5). Given that play fosters the social reciprocity, self-management and creative thought processes deemed necessary for school readiness, time spent playing provides essential preparation for learning receptivity and responsiveness.

Consistent with these views, early childhood education programs have been historically grounded in child-directed play experiences with a primary focus on developing social relationships, approaches to learning, and self-regulation (Burchinal et al., 2008). Play as the focus of early childhood education remained essentially unchallenged in the United States until 1983, when the National Commission on Excellence in Education issued a report entitled *A Nation at Risk: The Imperative for Education Reform*. This Presidential-commissioned report described education in the United States as “a rising tide of mediocrity” citing American children’s poor academic performance, rampant illiteracy among adults and teens and declining scores on standardized achievement tests measuring academic competency. The authors recommended sweeping educational reforms with higher expectations of student performance and rising academic achievement test scores as the cornerstone of a stronger American education system (National Commission on Excellence in Education, 1983). Congress responded by enacting educational reforms, beginning with *America First* (1991), a comprehensive education reform act aimed at improving school readiness.

*America First* defined school readiness along five domains: physical health and wellbeing, social-emotional development, language development, general knowledge and cognition, and approaches to learning. Most states evaluated school readiness by measuring general knowledge and cognition, placing less focus on the other four domains. Subsequent educational reform legislation, No Child Left Behind (NCLB; 2002) and Race to the Top-Early Learning Challenge (RTTT-ELC; 2013), further cemented administration of standardized assessments prior to kindergarten entry. This represented a substantial shift from formative assessments of early childhood education, where teachers evaluated children over time (Hustedt et al., 2018).

As early childhood educators navigated increasing pressures for proficiency in early literacy and numeracy, the content of many early childhood education programs emphasized academic content (Fleer, 2021; Nicolopoulou, 2010; Taylor & Boyer, 2019). However, academic instruction in preschool is controversial; some early childhood educators have expressed concern about the developmental appropriateness of direct instruction practices (NAEYC, 2017; Zosh et al., 2017). This philosophical split left some early childhood programs grounded in play-based, child-directed experiential learning while others emphasized direct academic instruction. Durkin et al. (2022) stated that direct instruction in early childhood education produces short-term gains in constrained literacy skills (e.g., recognizing the alphabet), but not the unconstrained literacy and numeracy skills associated with long-term academic success (e.g., comprehension, problem-solving). This suggests that content-heavy preschool curricula most aligned with school readiness assessments may fail to capture the foundational aptitudes children need for academic achievement.

School readiness is a complicated and nuanced concept; children need more than general knowledge and academic skills to thrive at school. Hustedt et al. (2018) found that kindergarten teachers favored non-academic skills as most valuable: caring for personal needs, exhibiting self-control, communicating needs and preferences, modifying behavior and interacting cooperatively. Pistorova and Stutsky (2018) further argued that the twenty-first century learner must be fluent with “critical thinking, communication, collaboration and creativity” (p. 495); these are learned through capitalizing on children’s natural curiosity and inquisitiveness. Zosh et al. (2017) stated that optimal learning occurs within a playful context where children experience joyfulness through engagement in meaningful activities. Play-based learning allows children to develop and test theories and make new discoveries, thus expanding their capacity for problem-solving and construction of higher-order conceptual schemas (Fleer, 2021; Sim & Xu, 2017). Finally, peer play and social relationships have been associated with higher levels of global teacher-rated kindergarten competence including following directions, academic receptivity, self-regulation, and cooperative interactions (Eggum-Wilkins et al., 2014).

Johansson and Samuelsson (2006) argued that play and learning in young children are integrated, and researchers must seek to understand the relationship between play and learning rather than dichotomize them with false distinctions. Nilsson et al. (2018) advocated for a *play-as-learning* approach to early childhood education, where learning “is not just understood in the narrow cognitive sense…but more broadly as transformations driven by different kinds of experiences that lead to sustained change” (p. 232). This suggests a need for research aimed at explicating the ways in which play contributes to readiness for formal education. Therefore, the purpose of this paper is to describe how teachers and parents interpreted the school readiness of four children as they navigated kindergarten after three or more years of play-based early childhood education at a Reggio Emilia-inspired school.

## Methods

This manuscript is part of a larger, longitudinal study exploring how children fared as kindergarteners following play-based early childhood education, in partial fulfillment of the requirements for the degree of Doctor of Philosophy in Occupation and Rehabilitation Science at Colorado State University for first author LF. Data were collected from September 2020 through May 2021. This study was approved through the Colorado State University Institutional Review Board (approval 19-9519H).

### Research Design

We used Yin’s (2018) cross-case study approach to synthesize findings from the cases. We collected data at the onset, midpoint, and conclusion of the kindergarten year. Each data cache informed subsequent data collection.

### Participants

We recruited participants from a Reggio Emilia-inspired play-based early childhood center in Northern Colorado. Reggio Emilia is a highly regarded approach to early childhood education where children collaborate with their teachers to explore their interests through projects which are thoughtfully designed and documented (McNally & Slutsky, 2017). We enrolled four participant clusters in the summer preceding the child’s kindergarten enrollment; each cluster ideally included a parent and a kindergarten teacher associated with an individual child. Mothers were the parent informant for all four clusters and provided contact information for their child’s kindergarten teacher. Three of four kindergarten teachers participated; one declined due to concerns with the COVID-19 pandemic-affected school year.

Kindergarten teachers represented three different curricular foci: Core Knowledge, International Baccalaureate. and Science, Technology, Engineering and Math (STEM). The research team deemed dissimilar kindergarten foci desirable to offer variation in kindergarten experience, but this was not a participant requirement. Table 1 contains a description of cluster members organized by child.

**Table 1:** Descriptors and characteristics of participant clusters (code names used)

Child pseudonym	Addy	Isaac	Leah	Nadine
Gender	Female	Male	Female	Female
Age at initial interview	5 years, 7 months	6 years, 1 month	5 years, 6 months	5 years, 6 months
Birth order	2nd of two siblings	1st of two siblings	2nd of two siblings	2nd of two siblings
Teacher participants’ time teaching	28 years	14 years	11 years	Unknown
Curricular focus of school	Core knowledge	Science, technology, engineering and math	International baccalaureate	International baccalaureate

#### Pandemic Influence

Public health orders related to the COVID-19 pandemic affected the kindergarten year. Over half of the kindergarten year occurred through remote learning and all in-person school days were affected by public health orders. Requirements of face masks, social distancing, and isolated classroom cohorts limited participation in specials, lunchroom, recess, and group learning experiences. Teacher participants described challenges translating learning activities to remote platforms, altering the scope and sequence of the curriculum, and emotional and behavioral challenges engaging students during in-person learning.

Public health orders also altered data collection as visitors were not allowed in public schools. We adapted to these restrictions by completing home visits during remote learning days, requesting work samples and test scores from participants, and using video conferencing for most interviews.

### Procedures

**Fig. 1:**
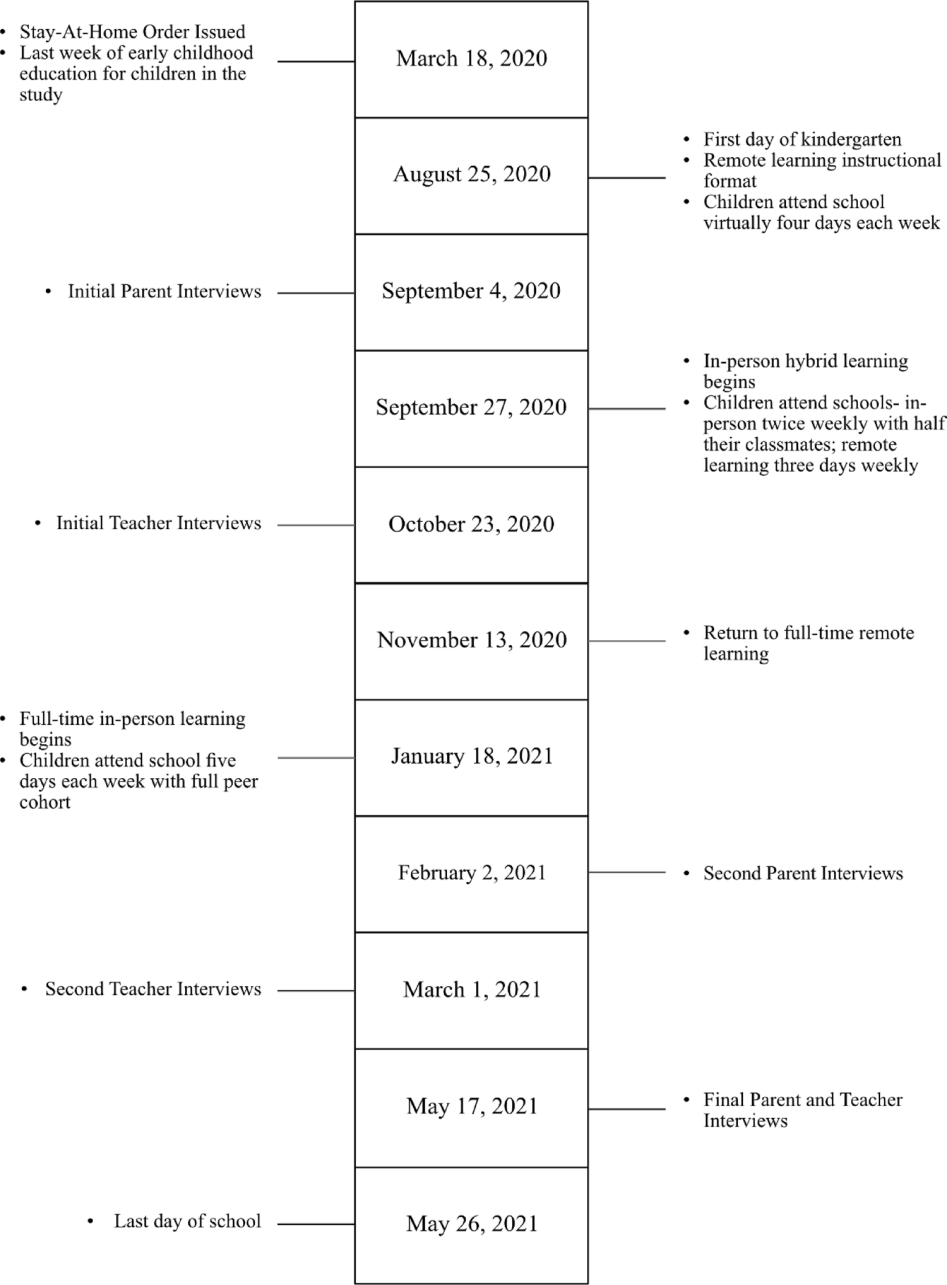
Timeline of public health orders related to COVID-19, district-wide instructional format shifts, and participant interview schedule

We used semi-structured interviews with participants as the primary data source. Each participant completed three in-depth semi-structured interviews lasting approximately one hour per encounter. The interviews coincided with the start of the school year and planned instructional format shifts. We recorded all interviews; a professional service transcribed them verbatim. See Fig. 1 for an illustration of the timing of interviews alongside of the start and end of the school year and district-wide instructional shifts.

We developed separate but parallel interview protocols for the participant groups prior to each interview. Some questions were written for both groups; others were written only for parents or teachers. We wanted to capitalize on each participant’s unique vantage point, while allowing for comparisons of perspectives within and across cases. For example, we asked both parents and teachers about the child’s school readiness based on Colorado’s early learning standards. We asked teachers to describe how the child fit within their classroom expectations and parents to describe how their child had grown over the course of the school year. See Table 2 for interview protocol examples.

**Table 2:** Sampling of Interview Protocol Questions by Participant Group

Interview Foci	Sample Question
School readiness along Colorado early learning standards (2016)	The State of Colorado (2016) describes six early learning development areas that promote readiness for kindergarten. How would you describe the readiness of [child] along these lines when s/he began kindergarten last fall?^a^
	Academic knowledge such as letter and number recognition,
	Overall health and development such as physical well-being and motor development,
	Social emotional development such as forming healthy relationships,
	Cognition such as attention and problem solving,
	Language and comprehensions such as verbal communication skills and
	Approaches to learning such as varied interests in topics and perseverance in accomplishing goals
Meeting classroom teachers’ expectations for kindergarteners	In completing your on-going formative and summative assessments and progress monitoring over the past school year, how well would you say [child] fit within your expectations for kindergarteners in your classroom this past year?^b^
	When you think about the children in your classroom this year, what stands out to you about [child] as a learner or classmate?^b^
Understanding primary contributors to the child’s growth over the kindergarten year	Now that you know what their kindergarten year entailed, how do you think play-based education contributed to [child’s] kindergarten experience?^c^

^a^Asked of all participants

^b^Asked of teachers only

^c^Asked of parents only

In the case of the child whose teacher did not participate (Nadine), we used the same parent interview protocol with her mother as we did with the other parent participants. Nadine’s mother provided work samples and school-generated reports. We blended Nadine’s data within the cross-case analysis as appropriate, acknowledging that we had half the data for her as other participants.

### Data Analysis

Congruent with Yin’s (2018) case-based approach, we analyzed each participant interview upon its conclusion and merged the interviews by case to complete the initial within-case analysis. Once we identified themes from an individual case, we looked for replication across cases, remaining sensitive to congruences and differences as we identified patterns. By comparing patterns across cases, we constructed common themes.

#### Within-Case Analysis

In the initial phase of analysis, first author LF read each transcript multiple times for familiarity, documented any reactions and biases, and listed all topics emphasized by the participants for each case. In the second phase, these preliminary topics provided an organizational structure for grouping the data into conceptual clusters. In the third phase, authors LF, PS and AB systematically worked through groupings and developed initial codes with extrapolated, verbatim text illustrating the themes under consideration. In the fourth phase, author LF defined and described the themes from the individual cases and selected representative quotes for illustration.

#### Cross-Case Analysis

**Fig. 2:**
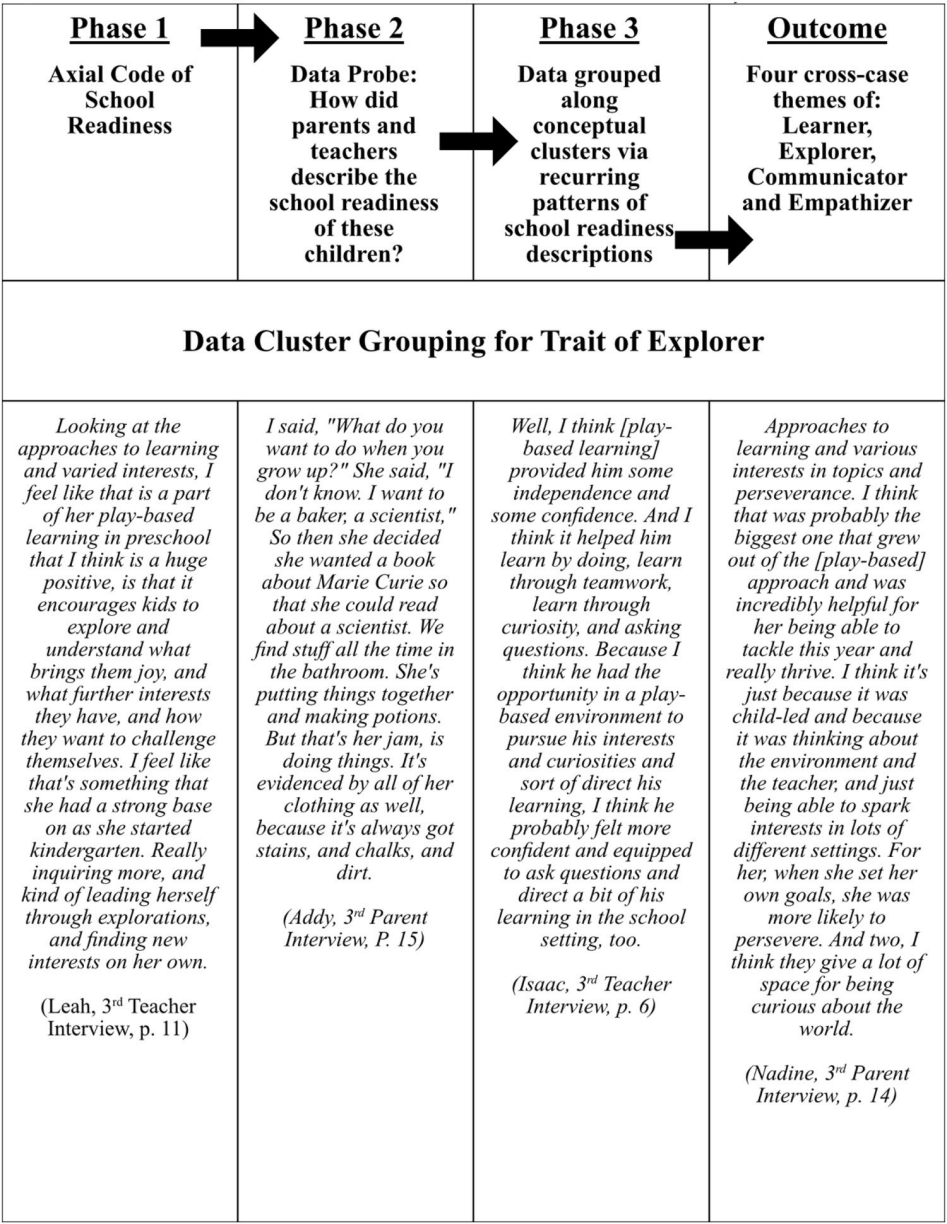
Explorer theme construction: within-case analysis to cross-case analysis

Cross-case analysis occurred after individual case analyses. First, author LF reduced the data set to focus on the most significant conceptual clusters; these became axial codes linking the data across cases. For this manuscript, school readiness was the axial code of interest (kindergarten performance and adaptation were the other axial codes and will be the foci of subsequent papers). In the second phase, author LF operationalized school readiness into a probing question (*How are parents and teachers describing the school readiness of these children?*), which guided the cross-case analysis. Finally, authors LF, PS and AB constructed cross-case themes using inductive coding to extrapolate the major descriptors of school readiness common to all cases. First author LF served as the primary coder, PS was the second coder and both PS and AB engaged in ongoing conversations with LF to define, clarify, and construct the final themes. These themes were then presented to the entire research team for discussion until consensus was reached. See Fig. 2 for a graphic illustrating this process.

### Quality Assurance

We took many steps to safeguard the quality of the study data, as well as the data analysis. We remained engaged with participants for a full academic year which allowed for data triangulation and opportunities for member checking. We practiced highly disciplined subjectivity through data trail audits, explicit links between interpretation and participant excerpts, and constant comparative analysis within and across cases (Yin, 2018). We held recurring meetings to discuss the findings and arrive at consensus with all members of the research team.

#### Positionality

Author LF supervised occupational therapy students completing internships at the play-based early childhood center where the children were enrolled prior to entering kindergarten. Author PS has expertise in qualitative research methodology but had not worked with early childhood research prior to this study. Author AL is an early childhood education teacher educator and coached teacher candidates at the research site. Author KR was the Executive Director of the center during the time when this study occurred. Author AB is an expert in play and has many years of school-based experience as an occupational therapist and researcher.

## Findings

Participants believed the children were well prepared for kindergarten. Four themes were identified through the data analysis process that illustrate their school readiness: *learners, explorers, communicators, and empathizers*. See Table 3 for full definitions of each theme.

**Table 3:** School readiness theme definitions, descriptions, and illustrations

Theme	Definition	Description	Illustrative Quotes
Learners	Expressing beliefs or sharing observations illustrating the child’s receptivity to acquiring new knowledge	Stories, perceptions, or observations of how the child engaged in or responded to academic learning	What I could tell is that he was ready to learn. He was really engaged with letters and numbers, and he was eager. He was excited. It was the perfect time to start teaching him how to read and write… (Isaac, 3rd Teacher Interview, p. 13)
Explorers	Expressing beliefs or sharing observations illustrating the child’s approach to learning, inquisitiveness, or ability to drive their learning	Stories, perceptions, or observations of the child’s creativity, inquisitiveness, or initiative; also includes descriptions of how the child approached learning	She wasn’t coming in just wanting to follow instructions exactly, which I think is a great way to approach learning. So, I think for her, she kept that creative approach. (Nadine, 3rd parent Interview, p. 19)
Empathizers	Expressing beliefs or sharing observations illustrating the child’s capacity for empathy with others	Stories, perceptions, or observations of the child’s interactions where they demonstrate compassion, insight, or thoughtfulness	She’s just very thoughtful in how she interacts with others. Really high skills with empathizing with others. And I feel like that’s something that you don’t often see in kindergartners.(Leah, 3rd Teacher Interview, p. 5)
Communicators	Expressing beliefs or sharing observations illustrating the child’s self-advocacy, conflict management or relationship skills	Stories, perceptions, or observations of the child seeking relationships, managing conflict, or negotiating with others to solve social dilemmas	She knows how to listen to directions, but also if something isn’t quite going her way, she has been known to say how she feels. I have never heard her, necessarily, argue with someone about something. She seems to handle conflict pretty well. (Addy, 3rd Teacher Interview, p. 17)

### Theme 1: Learners

We defined *learner* as a child who is highly receptive to acquiring new knowledge. Data within this theme described children’s responses to classroom instruction and teacher feedback. Teachers reported that the children arrived at kindergarten ready to learn and were quick to integrate new knowledge and concepts. They noted well-developed language, eagerness for learning, and willingness to embrace feedback and challenge. Isaac’s teacher described him by stating.

What I could tell is that he was ready to learn. He was really engaged with letters and numbers, and he was eager. It was the perfect time to start teaching him how to read and write… (Isaac, 3rd Teacher Interview, p. 13)

Addy’s teacher described her by saying.

She knew about half of her letter names and sounds, and that's actually a little ahead of the game for a lot of kiddos coming into kindergarten. She has a good sense of what word would make sense when she gets to a word she doesn’t know…it’s like it’s innate with her. So that tells me that she had a very good background in those phonemic awareness skills… (Addy, 3rd Teacher Interview, p. 4)

Teachers described the children as self-directed, which heightened their readiness to retain new information and engage in academics. Leah’s teacher described her, saying, “I feel like [self-direction] is something that she had a strong base on as she started kindergarten. Really inquiring more, and kind of leading herself through explorations, and finding new interests on her own.” (Leah, 3rd Teacher Interview, p. 11). Nadine’s mother described her inquisitiveness by saying “Being able to spark interests in lots of settings was incredibly helpful for her to tackle this year and really thrive” (Nadine. 3rd Parent Interview, p. 14).

Teachers also expressed how the ability to persist through challenge and apply feedback benefitted the children. Leah’s teacher described her by saying:

She understands challenge, and she understands the benefit from challenge. She doesn’t shy away, because she sees how challenge benefits her as a learner and a problem solver. She understands what her job is as a student and wants to do her very best with that. (Leah, 3rd Teacher Interview, p. 12)

Isaac’s teacher described him as a responsive learner who takes feedback and applies teachings in a variety of contexts. “He’s a quick learner. He will apply what I’ve asked him to change and take it in a direction I’d like him to go*.” (Isaac, 3rd Teacher Interview, p. 7).*

### Theme 2: Explorers

While the *learner* theme centered on how a child reacted to their classroom experiences, the *explorer* theme centers on how a child approached learning and applied new knowledge. We defined *explorer* as a child who is inquisitive, creative and takes initiative in seeking and expanding upon their learning experiences. Parents and teachers saw inquisitiveness as a strength of all the children. They shared stories of the children’s creativity and open-mindedness. Nadine’s mother described how she used exploration to follow an interest in the Fibonacci rule of spirals. She drew pictures of the pinecones she found in their backyard, carefully documenting their shape and composition.

I think [varied interests] was probably the biggest one that grew out of [play-based learning] and was incredibly helpful for her being able to tackle this year and really thrive. I think it’s just because it was child-led and because it was thinking about the environment and the teacher, and just being able to spark interests in lots of different settings. You just figure out your own goals and you’re trying to set them yourself. For her, when she set her own goals, she was probably more likely to persevere. And two, I think they give a lot of space for being curious about the world. (Nadine, 3rd Parent Interview, p. 27)

Addy’s mother told a story of how Addy was inspired by a book about Marie Curie and subsequently spent time mixing potions in the bathtub. She felt that play-based learning instilled a curiosity and desire to learn through exploration and discovery in Addy.

So, like I said, “What do you want to do when you grow up?” She said, “I don’t know. I want to be a baker, a scientist.” So, then she decided, when they were out at the store, she wanted a book about Marie Curie so that she could read about a scientist. And we find stuff all the time in the bathroom. She’s putting things together and making potions. But that’s her jam, is doing things. But it’s evidenced by all of her clothing as well, because it’s always got stains, and chalks, and dirt. So, she’s in there. (Addy, 3rd Parent Interview, p. 15)

Isaac’s mother spoke of the connection she observed between exploration, inquisitiveness, and confidence and how this impacted Isaac’s engagement with learning in kindergarten.

Well, I think [play-based learning] provided him some independence and some confidence. And I think it helped him learn by doing, learn through teamwork, learn through curiosity, and asking questions. Because I think he had the opportunity in a play-based environment to pursue his interests and curiosities and sort of direct his learning, I think he probably felt more confident and equipped to ask questions and direct a bit of his learning in the school setting, too. (Isaac, 3rd Parent Interview, p. 6)

Leah’s mother described how play-based learning contributed to Leah’s open-mindedness about the world and her willingness to take initiative with learning.

Because of her play-based background, I really think that she’s been in a place where she’s been able to be that much more open-minded. I think the focus on explorative play has been crucial for learning different ways of perceiving and understanding the world around her and fostering her as an innate learner and leader of her own development. And I think it would have been much more difficult to have had that type of growth in a different type of setting. *(Leah, 3rd Parent Interview, p. 9)*

Participants also attributed the children’s varied approaches to learning to play-based experiences. Nadine’s mother described how Nadine continued to draw on the creativity fostered through play-based learning in approaching her academics.

She wasn’t coming in just wanting to follow instructions exactly, which I think is a great way to approach learning. Because I know sometimes if you just think about following the steps of something to get the A or something like that, to me it kind of undermines the joy of learning. So, I think for her, she kept that creative approach. (Nadine, 3rd parent Interview, p. 19)

### Theme 3: Communicators

We defined *communicator* as a child who demonstrates self-advocacy, conflict management or relationship-building skills. Data within this category shows how the children expressed themselves and established social connections. Parents and teachers described the children as in-tune with their peer cohorts, well-versed in considering other perspectives and able to describe and advocate for their needs. Teachers noted that social communication was a struggle for many children entering kindergarten during the 2020–2021 school year, but felt these children were effective at establishing social connections and cultivating relationships with peers and adults.

Addy’s teacher spoke about her “Friday Fun’ group where kindergarteners were allowed to free play with vintage toys and art supplies for an hour in the classroom.

This year, she had to remain constantly present during this time because the children were unable to share toys and develop a play schema together. Addy, however, was always able to find something to do and negotiate with others for turn taking and sharing of materials.

I feel like for a kindergartner she came in knowing a lot of the social cues and how to move around a classroom, how to manage listening to directions, but also if something isn’t quite going her way, she’ll either go do something else or say how she feels. I have never heard her, necessarily, argue with someone about something. She seems to handle conflict pretty well, which, I didn’t really teach her that. (Addy, 3rd Teacher Interview, p. 16)

Leah’s mother described how Leah used her negotiation skills to intervene during a potentially challenging situation when Leah and her friends all wanted to play dress-up with the same butterfly cape.

I think that where [play-based learning] was really helpful for her was in learning to speak up for difficult situations. To me, watching her negotiate with those friends how much time each child is going to have with this really great outfit... I think that that has been evident to me that she really does have a way of kind of owning her feelings and stepping up and saying something. It’s just hard to know of course, but I think the skills that she has now, in terms of her confidence, really grew out of negotiating with others in a play-based environment. (Leah, 3rd Parent Interview, p. 10)

Isaac’s mother described how Isaac’s ability to communicate was helpful in navigating the emotions of his kindergarten year.

He used to talk about when he was struggling. He will say, “I’m feeling sad right now, I am feeling upset right now.” He’ll just say it. Sometimes we know it’s coming, and sometimes we didn’t. I think that was a real advantage for this year in particular. (Isaac, 3rd Parent Interview, p. 20)

Leah’s teacher described a scenario where Leah sought out a weekly meeting with the Assistant Principal to discuss life events, which she chose over her recess time.

I have come to understand how important personal connection is for [Leah]. And so, she is amazing with interactions and relationships with kids, but she’s clearly somebody who seeks that connection with other adults. And I talked about her in class just being such a great listener with friends and classmates. But it’s all around. She just has genuine interest in learning from other people’s stories. (Leah, 3rd Teacher Interview, p. 10)

### Theme 4: Empathizers

We defined *empathizer* as a child who expresses compassion towards others, and data within this theme represent how children engaged thoughtfully with their classmates. Teachers especially described scenarios where these children exceeded their expectations for social reciprocity and initiating acts of kindness. Addy’s teacher described Addy by saying, “all the kids at this point have been taught how to be kind but to see a child actually notice a friend who is alone and do something about it is quite remarkable.”

Leah’s teacher also noted Leah’s exceptionally well-developed empathy and felt this was a result of immersion in play-based learning.

Social emotional development, I feel like she’s at the top of her class with that. She’s just very thoughtful in how she interacts with others. Really high skills with empathizing with others. And I feel like that’s something that you don’t often see in kindergartners. It takes a lot of development, but I feel like that’s something that [Leah] had walking into kindergarten. (Leah, 3rd Teacher Interview, p. 5)

Isaac’s teacher spoke of Isaac’s awareness of others’ emotions, especially classmates with disabilities. She stated that Isaac persists in these relationships because he wants to help these children succeed.

He sees these good strengths in the other kids. And even when it’s bothering him, he wants to be there to work through the issues, kind of like just an old soul in a sense that he kind of has this awareness and then the verbal skills to help this child through the process. (Isaac, 3rd Teacher Interview, p. 18)

Nadine’s mother spoke of how Nadine reacted to children who were emotionally distraught by extending compassion in emotionally charged situations.

And I feel like there are times when our kids have been upset, for sure, but we’ve heard what seems like trauma. Kids throwing chairs in the classroom and stuff that I don’t remember ever happening when I was in school. And our kids talk about it and say, someone had kind of a rough day or like lost control of his emotions, which I feel like was an incredible way to put it. (Nadine, 3rd Parent Interview, p. 24)

## Discussion

The purpose of this qualitative cross-case study was to describe the school readiness of four 5-year-old children entering kindergarten following play-based learning at a Reggio Emilia-inspired early childhood center. Participants described the children as learners, explorers, communicators, and empathizers, and believed the children were ready for kindergarten, engaged in creative thought, were responsive to novel learning experiences and classroom expectations, were well-versed in social communication and oriented towards connecting with others.

Literature addressing play and learning suggests children entering kindergarten following play-based early childhood education should excel in three key areas of school readiness. First, they should be excellent thinkers and learners who are curious about the world around them, capable of driving their learning and exploring their interests. Second, they should be fluent in emotional intelligence and adept at modifying their actions in accordance with group norms and social feedback. Finally, they should be confident in relationship building, readily forging connections with peers and adults and executing social reciprocity with ease (Harrington et al., 2020; Immordino-Yang et al., 2019; Taylor & Boyer, 2019).

Zosh et al. (2017) stated that children need to be ready to develop deeper conceptual understandings of the knowledge they acquire in school to be proficient learners and contributors to their classroom. Play provides recurring and novel opportunities for children to think deeply about the concepts they are learning, thus fostering complexity of thought and the development of complex schemas. Our findings offer evidence that play-based learning was effective in nurturing the thinking and understanding of the children who were the focus of this study. From Nadine’s careful recording of the pinecone shapes she observed in her backyard as she pondered the Fibonacci rule of spirals to Addy’s potion-making in the family bathtub, the children we studied took the information they learned and used it to investigate hypotheses and record their observations of nature. These children embodied the process of learning through self-directed exploration, which allowed them to make new discoveries and use the knowledge they acquired to serve a purpose.

Nilsson et al. (2018) championed play-as-learning because of the transformational changes play fosters within children as they enact societal roles and norms. Through play, children explore that which they have learned and discern how to apply or revise their knowledge in collaboration with others. Because play challenges children to leverage existing knowledge to solve social dilemmas, they become increasingly fluent in the process of reading social feedback and advocating for their position. Leah embodied this when she led negotiations among friends to determine acceptable rules for sharing a prized cape during dress-up play. Isaac’s teacher illustrated this through her observations of Isaac using his social capitol to include classmates with disabilities within the social hierarchy. Addy’s teacher observed this when she described Addy’s competency with reading social cues and following directions. In all cases, these children showed an ability to alter their behavior in response to social expectations.

Learning is inherently social, and children who are emotionally responsive have an easier time forming social connections (Harrington et al., 2020; Immordino-Yang et al., 2019). Establishing positive social relationships advantaged these children by allowing them to tap into the knowledge of others and learn collaboratively through group exploration. Addy’s teacher illustrated this when she reflected on Addy approaching a child sitting alone on the playground and inviting her to play. She noted that seeing Addy notice and extend an invitation to the solitary child was beyond the social maturity she expected of kindergarteners. Leah’s pursuit of a weekly meeting with her Assistant Principal also showed her prowess in building and sustaining relationships with others. Leah’s teacher’s description of her desire to “learn from the stories of others” shows the extent to which these children valued social connections and used their relationships with others to obtain new understandings.

These children’s proficiency in building relationships and social connections likely contributed to their success in kindergarten in many ways. One of our findings that was not explicit in play-based learning literature was the extent to which these children embraced challenges and persisted through difficulty. Burchinal et al. (2008) described the student–teacher relationship as the most impactful element of early learning in young children. The warm and trusting relationships these children fostered with their teachers likely contributed to their willingness to push themselves academically and accept constructive feedback to make progress as kindergarteners.

Through immersion in child-directed, exploratory learning, these children arrived at kindergarten with well-established schemas for acquiring and using new knowledge. They came from a background where they were encouraged to follow their interests and have voice in both the content and format of their educational experiences. Part of being the driver of your own learning is that you become adept at solving problems. This likely advantaged the children in that they saw themselves as co-creators of knowledge, capable of making meaningful contributions to their learning experiences. This was reflected when Leah’s teacher said that Leah showed “many ways of learning” and in Nadine’s mother’s comment that Nadine “kept that creative approach” when she was exposed to direct instruction in academic concepts and teacher-provided learning materials.

Our findings align with holistic views of education, given that the parents and teachers described these children as fluent with the non-constrained aspects of school readiness (Durkin et al, 2022; Taylor & Boyer, 2019). Their ability, however, to use social language and draw connections among concepts allowed them to master the more constrained elements of learning with persistence and enthusiasm. For example, Isaac was described as having the language skills to work through interpersonal problems with his peers and share his emotions with both his teacher and his mother, which helped him navigate social conflict and process his feelings of sadness or frustration. Because he was able to process emotional experiences in a supportive and productive manner, he was likely more available and present for classroom instruction.

The socioemotional school readiness attributes favored by teachers as preparing children for school are difficult to quantify, though parents and teachers readily observed them in the children studied. Time spent in play-based learning, where these children were empowered to follow their interests and actively collaborate with teachers and peers to co-create and lead their learning experiences, seemingly instilled within them a deep understanding of the learning process. These children arrived at kindergarten having established robust approaches to learning, which may have contributed to their competency with acquiring new knowledge and responding to various learning environments and teaching strategies. The children saw learning as an opportunity to pursue their interests, and as Nadine’s mother observed, when children set their own goals, they are much more likely to persevere through challenge.

The children who were the focus of this study faced many challenges beyond what would be expected during a typical school year and had access to fewer of the supports normally offered to buffer them against those stressors. The study of kindergarten transition during a time of high stress and uncertainty was likely, and perhaps surprisingly, an asset to our research, as the intensity of experiences faced by these children illuminated their readiness in unique and varied ways. For example, having to navigate changes in instructional format revealed participants’ prowess with cognitive flexibility and persistence, while learning from home showcased their ability to drive their interests and explore topics.

## Conclusions

Early childhood educators and education policy makers face two fundamental questions when evaluating the readiness of children to enter kindergarten and benefit from formal education: (1) What foundational aptitudes best prepare children for long-term educational success? and (2) What pedagogical practices provide the optimal learning context for children prior to entering kindergarten? Children have many different life experiences that contribute to their readiness for kindergarten and perhaps it is more important to think about *how* various experiences prepare children for kindergarten rather than *what* experiences prepares children for kindergarten.

The children who were the focus of this study were very well prepared for successful kindergarten experiences by their time in play-based education. School readiness is a mindset that allows children to acclimate to and benefit from group learning contexts, requiring a certain adaptability and responsiveness to novelty and change to be successful. Perhaps play’s secret is its novelty and variation in challenging children to apply the knowledge they have and develop new understandings as situations become increasingly complicated; thus, preparing them for the challenge of kindergarten.

## Limitations

The nature of qualitative research is to explore participant experiences within their natural context through an in-depth, rich account of a relatively small number of participants (Yin, 2018). Congruent with cross-case study and qualitative research paradigms, the descriptions of these children’s preparedness for school were derived from the collective perspectives of their mothers and kindergarten teachers and were not compared with other measures of school readiness such as school-administered assessments (which were cancelled because of the pandemic). The children who were the focus of this study were well-prepared for kindergarten following play-based learning and the participants’ descriptions helped to deepen readers’ understanding of the role of play in that preparation. Nonetheless, there are many influences on children’s early development, and we cannot definitively say how these children’s experiences would compare with children entering kindergarten from a non-play-based setting.
